# Supplementary material for: Role of the NUDT Enzymes in Breast Cancer
Source: Int J Mol Sci. 2021 Feb 25;22(5):2267. doi: 10.3390/ijms22052267 (PMC7956304; doi:10.3390/ijms22052267)
Supplement: Supplementary file 1 [file ijms-22-02267-s001.pdf]

Supplementary Material.

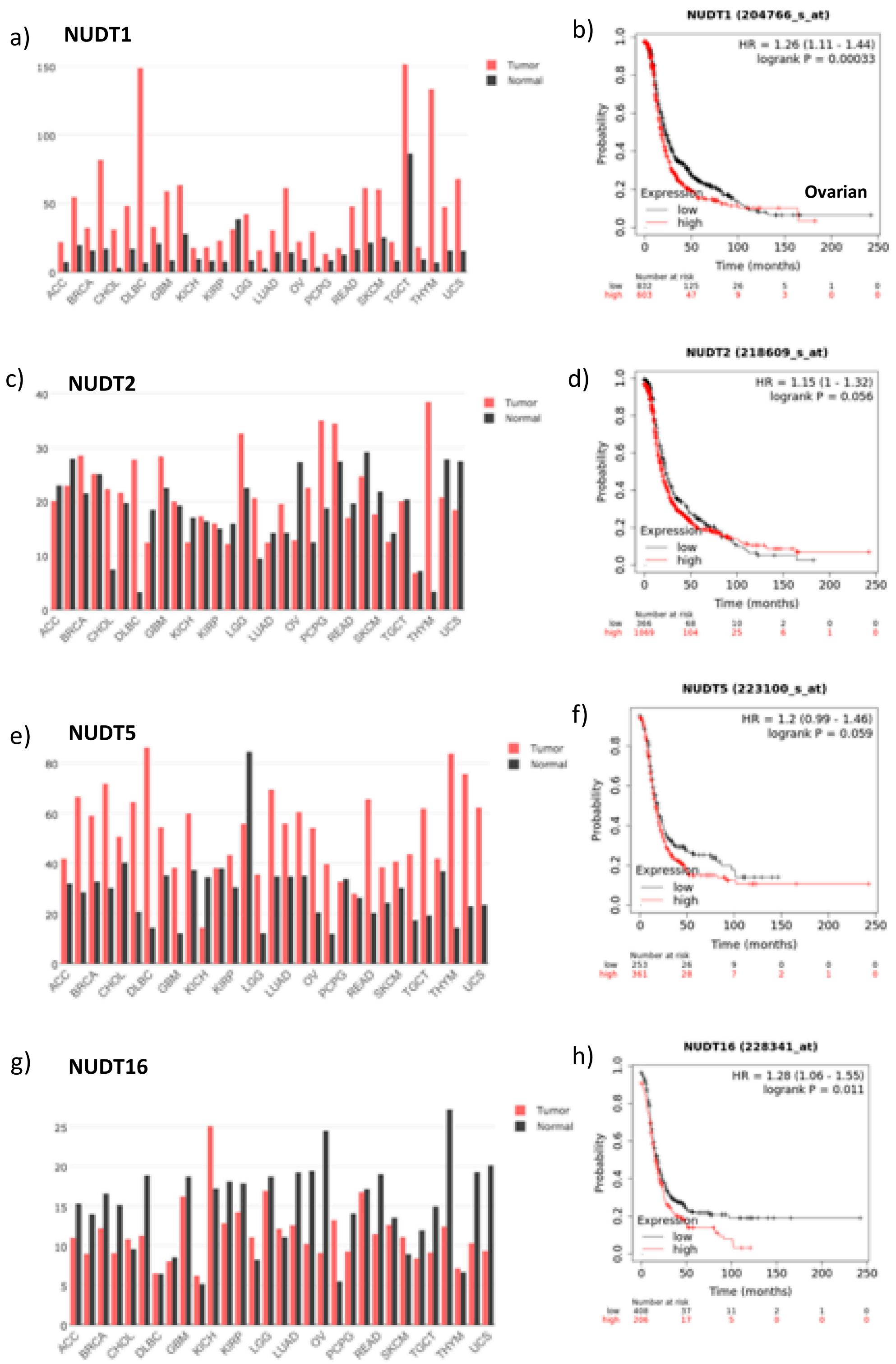

Supplementary Figure 1. Expression levels of NUDT family members in different cancer types. a) Expression level of NUDT1 in TGCA cancer datasets. b) Overall survival in ovarian cancer based on patient stratification using expression levels of NUDT1. c) Expression level of NUDT2 in TGCA cancer datasets. d) Overall survival in ovarian cancer based on patient stratification using

expression levels of NUDT2. e) Expression level of NUDT5 in TGCA cancer datasets. f) Overall survival in ovarian cancer based on patient stratification using expression levels of NUDT5. g) Expression level of NUDT16 in TGCA cancer datasets. h) Overall survival in ovarian cancer based on patient stratification using expression levels of NUDT16.

Supplementary Table 1. Full data mining for NUDT1, 2, 5 and 16 in different cancer types. Data information regarding the dataset ID, cancer type, endpoint measured, cohort ID, p value and corrected p value based on expression level of NUDT enzyme indicated.

	Dataset	Cancer Type	Endpoint	Cohort	p value	p value
NUDT1	GSE31210	Lung cancer	Relapse Free Survival	NCCRI	0.000000	0.000022
	GSE11121	Breast cancer	Distant Metastasis Free Survival	Mainz (1988-1998)	0.000002	0.000122
	GSE9893	Breast cancer	Overall Survival	Montpellier, Bordeaux, Turin (1989-2001)	0.000005	0.000227
	GSE9195	Breast cancer	Relapse Free Survival	GUYT2	0.000008	0.000351
	GSE12276	Breast cancer	Relapse Free Survival	EMC	0.000013	0.000574
	GSE13507	Bladder cancer	Disease Specific Survival	CNUH	0.000013	0.000579
	GSE19234	Skin cancer	Overall Survival	NYU	0.000024	0.000990
	GSE4271-GPL96	Brain cancer	Overall Survival	MDA	0.000047	0.001787
	GSE31210	Lung cancer	Overall Survival	NCCRI	0.000102	0.003558
	GSE4922-GPL96	Breast cancer	Disease Free Survival	Uppsala (1987-1989)	0.000134	0.004534
	GSE12417-GPL96	Blood cancer	Overall Survival	AMLCG (1999-2003)	0.000142	0.004754
	GSE3143	Breast cancer	Overall Survival	Duke	0.000219	0.006948
	GSE1456-GPL96	Breast cancer	Disease Specific Survival	Stockholm (1994-1996)	0.000397	0.011640
	GSE9195	Breast cancer	Distant Metastasis Free Survival	GUYT2	0.000398	0.011655
	E-TABM-346	Blood cancer	Overall Survival	GELA (1998-2000)	0.000754	0.020089
	jacob-00182-MSK	Lung cancer	Overall Survival	MSK	0.000801	0.021140
	GSE17536	Colorectal cancer	Disease Specific Survival	MCC	0.001632	0.038319
NUDT2	GSE4922-GPL96	Breast cancer	Disease Free Survival	Uppsala (1987-1989)	0.002233	0.049572
	GSE13507	Bladder cancer	Disease Specific Survival	CNUH	0.002081	0.046784
	GSE6532-GPL570	Breast cancer	Relapse Free Survival	GUYT	0.001859	0.042652
	GSE6532-GPL570	Breast cancer	Distant Metastasis Free Survival	GUYT	0.001859	0.042652
	GSE31210	Lung cancer	Overall Survival	NCCRI	0.001817	0.041865
	GSE8841	Ovarian cancer	Overall Survival	Milan (1992-2003)	0.001001	0.025507
	GSE31210	Lung cancer	Relapse Free Survival	NCCRI	0.000971	0.024853
	GSE9891	Ovarian cancer	Overall Survival	AOCS, RBH, WH, NKI-AVL (1992-2006)	0.000738	0.019734
	jacob-00182-CANDF	Lung cancer	Overall Survival	CAN/DF	0.000417	0.012119
	GSE5122	Blood cancer	Overall Survival	San Diego	0.000395	0.011576
	GSE17536	Colorectal cancer	Disease Specific Survival	MCC	0.000193	0.006232
	GSE1378	Breast cancer	Relapse Free Survival	MGH (1987-2000)	0.000182	0.005929
	GSE9893	Breast cancer	Overall Survival	Montpellier, Bordeaux, Turin (1989-2001)	0.000149	0.004959
	GSE17536	Colorectal cancer	Overall Survival	MCC	0.000109	0.003786
NUDT5	GSE13507	Bladder cancer	Disease Specific Survival	CNUH	0.000017	0.000732
	GSE31210	Lung cancer	Relapse Free Survival	NCCRI	0.000044	0.001678
	GSE1456-GPL97	Breast cancer	Relapse Free Survival	Stockholm (1994-1996)	0.000134	0.004531
	GSE1456-GPL97	Breast cancer	Disease Specific Survival	Stockholm (1994-1996)	0.000177	0.005786
	GSE13213	Lung cancer	Overall Survival	Nagoya (1995-1999, 2002-2004)	0.000192	0.006196
	GSE12417-GPL570	Blood cancer	Overall Survival	AMLCG (2004)	0.000228	0.007204
	GSE13507	Bladder cancer	Overall Survival	CNUH	0.000323	0.009736
	GSE9893	Breast cancer	Overall Survival	Montpellier, Bordeaux, Turin (1989-2001)	0.000369	0.010919
	GSE3494-GPL97	Breast cancer	Disease Specific Survival	Uppsala (1987-1989)	0.000456	0.013093
	GSE12276	Breast cancer	Relapse Free Survival	EMC	0.000615	0.016914
	GSE17260	Ovarian cancer	Progression Free Survival	Niigata (1997-2008)	0.001036	0.026262
	GSE12417-GPL97	Blood cancer	Overall Survival	AMLCG (1999-2003)	0.001125	0.028119
	GSE17260	Ovarian cancer	Overall Survival	Niigata (1997-2008)	0.001219	0.030073
	GSE1456-GPL97	Breast cancer	Overall Survival	Stockholm (1994-1996)	0.001526	0.036239
	GSE17710	ung cancer	Overall Survival	UNC	0.001893	0.043305
NUDT16	GSE4271-GPL97	Brain cancer	Overall Survival	MDA	0.002125	0.047609
	GSE9195	Breast cancer	Distant Metastasis Free Survival	GUYT2	0.001962	0.044583
	GSE4412-GPL97	Brain cancer	Overall Survival	UCLA (1996-2003)	0.001237	0.030439
	GSE14333	Colorectal cancer	Disease Free Survival	Melbourne	0.001222	0.030131
	GSE12276	Breast cancer	Relapse Free Survival	EMC	0.001063	0.026832
	GSE1456-GPL97	Breast cancer	Relapse Free Survival	Stockholm (1994-1996)	0.000920	0.023767
	GSE8894	Lung cancer	Relapse Free Survival	Seoul (1995-2005)	0.000820	0.021560
	GSE1456-GPL97	Breast cancer	Overall Survival	Stockholm (1994-1996)	0.000758	0.020184
	E-DKFZ-1	Renal cell carcinoma	Overall Survival	RZPD	0.000090	0.003178

Supplementary Table 2. Full data mining for all NUDT enzymes in breast cancer datasets. Data information regarding the dataset ID, cancer type, endpoint measured, cohort ID, p value and corrected p value based on expression level of NUDT enzyme indicated.

TYPE	ID_NAME	DATASET	CANCER TYPE	ENDPOINT	COHORT	N	MINIMUM P-VALUE	CORRECTED P-VALUE
GENE	NUDT10	GSE9195	Breast cancer	Distant Metastasis Free Survival	GUYT2	77	0.0117259	0.181885
GENE	NUDT10	GSE9195	Breast cancer	Relapse Free Survival	GUYT2	77	0.0186728	0.255217
GENE	NUDT10	GSE6532-GP	Breast cancer	Distant Metastasis Free Survival	GUYT	87	0.0218853	0.28545
GENE	NUDT10	GSE6532-GP	Breast cancer	Relapse Free Survival	GUYT	87	0.0218853	0.28545
GENE	NUDT10	GSE19615	Breast cancer	Distant Metastasis Free Survival	DF/HCC	115	0.0345718	0.389173
GENE	NUDT10	GSE1456-GP	Breast cancer	Relapse Free Survival	Stockholm (1994-1996)	159	0.0423414	0.443411
GENE	NUDT10	GSE1456-GP	Breast cancer	Disease Specific Survival	Stockholm (1994-1996)	159	0.0467768	0.471906
GENE	NUDT11	GSE9195	Breast cancer	Relapse Free Survival	GUYT2	77	0.00236804	0.0519996
GENE	NUDT11	GSE9195	Breast cancer	Distant Metastasis Free Survival	GUYT2	77	0.0034706	0.0708192
GENE	NUDT11	E-TABM-158	Breast cancer	Distant Metastasis Free Survival	UCSF, CPMC (1989-1997)	117	0.00721873	0.125971
GENE	NUDT11	GSE12276	Breast cancer	Relapse Free Survival	EMC	204	0.0130949	0.19737
GENE	NUDT11	E-TABM-158	Breast cancer	Overall Survival	UCSF, CPMC (1989-1997)	117	0.0136838	0.203856
GENE	NUDT11	E-TABM-158	Breast cancer	Relapse Free Survival	UCSF, CPMC (1989-1997)	117	0.0136838	0.203856
GENE	NUDT11	E-TABM-158	Breast cancer	Disease Specific Survival	UCSF, CPMC (1989-1997)	117	0.0311574	0.363335
GENE	NUDT11	GSE7390	Breast cancer	Overall Survival	Uppsala, Oxford, Stockholm, IGR, GUY	198	0.0350358	0.392584
GENE	NUDT11	GSE12093	Breast cancer	Distant Metastasis Free Survival	IO, NCI, TUM, CCF (1992-2000)	136	0.049112	0.48627
GENE	NUDT12	GSE9195	Breast cancer	Distant Metastasis Free Survival	GUYT2	77	8,75E+00	0.00311323
GENE	NUDT12	GSE12276	Breast cancer	Relapse Free Survival	EMC	204	0.000796034	0.0210337
GENE	NUDT12	GSE9195	Breast cancer	Relapse Free Survival	GUYT2	77	0.00248806	0.0541341
GENE	NUDT12	GSE19615	Breast cancer	Distant Metastasis Free Survival	DF/HCC	115	0.00423705	0.0830352
GENE	NUDT12	GSE1378	Breast cancer	Relapse Free Survival	MGH (1987-2000)	60	0.00636319	0.114266
GENE	NUDT12	GSE9195	Breast cancer	Distant Metastasis Free Survival	GUYT2	77	0.0163415	0.231959
GENE	NUDT12	GSE6532-GP	Breast cancer	Distant Metastasis Free Survival	GUYT	87	0.0338215	0.38361
GENE	NUDT12	GSE6532-GP	Breast cancer	Relapse Free Survival	GUYT	87	0.0338215	0.38361
GENE	NUDT12	GSE19615	Breast cancer	Distant Metastasis Free Survival	DF/HCC	115	0.04413	0.455101
GENE	NUDT12	GSE12276	Breast cancer	Relapse Free Survival	EMC	204	0.0445397	0.45774
GENE	NUDT12	GSE9195	Breast cancer	Relapse Free Survival	GUYT2	77	0.045521	0.464004
GENE	NUDT13	GSE4922-GP	Breast cancer	Disease Free Survival	Uppsala (1987-1989)	249	2,60E+00	0.0010577
GENE	NUDT13	GSE3143	Breast cancer	Overall Survival	Duke	158	0.000188499	0.00610415
GENE	NUDT13	E-TABM-158	Breast cancer	Distant Metastasis Free Survival	UCSF, CPMC (1989-1997)	117	0.000213139	0.00679327
GENE	NUDT13	GSE7849	Breast cancer	Disease Free Survival	Duke (1990-2001)	76	0.000347117	0.0103613
GENE	NUDT13	GSE7378	Breast cancer	Disease Free Survival	UCSF	54	0.000854055	0.0223196
GENE	NUDT13	GSE9195	Breast cancer	Relapse Free Survival	GUYT2	77	0.00106195	0.026802
GENE	NUDT13	GSE6532-GP	Breast cancer	Distant Metastasis Free Survival	GUYT	87	0.00124923	0.0306963
GENE	NUDT13	GSE6532-GP	Breast cancer	Relapse Free Survival	GUYT	87	0.00124923	0.0306963
GENE	NUDT13	GSE2990	Breast cancer	Distant Metastasis Free Survival	Uppsala, Oxford	125	0.00151227	0.0359763
GENE	NUDT13	GSE2990	Breast cancer	Distant Metastasis Free Survival	Uppsala, Oxford	125	0.00172228	0.0400566
GENE	NUDT13	GSE3494-GP	Breast cancer	Disease Specific Survival	Uppsala (1987-1989)	236	0.00197088	0.0447534
GENE	NUDT13	GSE2990	Breast cancer	Relapse Free Survival	Uppsala, Oxford	125	0.00776383	0.133217
GENE	NUDT13	GSE2034	Breast cancer	Distant Metastasis Free Survival	Rotterdam (1980-1995)	286	0.00836021	0.140971
GENE	NUDT13	GSE11121	Breast cancer	Distant Metastasis Free Survival	Mainz (1988-1998)	200	0.012097	0.186141
GENE	NUDT13	GSE2990	Breast cancer	Relapse Free Survival	Uppsala, Oxford	125	0.0136163	0.203118
GENE	NUDT13	GSE2990	Breast cancer	Relapse Free Survival	Uppsala, Oxford	62	0.0141904	0.209357
GENE	NUDT13	E-TABM-158	Breast cancer	Relapse Free Survival	UCSF, CPMC (1989-1997)	117	0.0181411	0.250017
GENE	NUDT13	E-TABM-158	Breast cancer	Overall Survival	UCSF, CPMC (1989-1997)	117	0.0181411	0.250017
GENE	NUDT13	GSE6532-GP	Breast cancer	Relapse Free Survival	GUYT	87	0.0209555	0.276899
GENE	NUDT13	GSE6532-GP	Breast cancer	Distant Metastasis Free Survival	GUYT	87	0.0209555	0.276899
GENE	NUDT13	GSE12276	Breast cancer	Relapse Free Survival	EMC	204	0.023337	0.298501
GENE	NUDT13	GSE12093	Breast cancer	Distant Metastasis Free Survival	IO, NCI, TUM, CCF (1992-2000)	136	0.0237845	0.302453
GENE	NUDT13	GSE2990	Breast cancer	Relapse Free Survival	Uppsala, Oxford	62	0.0240412	0.304705
GENE	NUDT13	GSE1456-GP	Breast cancer	Disease Specific Survival	Stockholm (1994-1996)	159	0.0277926	0.336483
GENE	NUDT13	GSE1456-GP	Breast cancer	Overall Survival	Stockholm (1994-1996)	159	0.0295836	0.350957
GENE	NUDT13	GSE7378	Breast cancer	Disease Free Survival	UCSF	54	0.0316946	0.36749
GENE	NUDT13	GSE9195	Breast cancer	Distant Metastasis Free Survival	GUYT2	77	0.0322741	0.371934
GENE	NUDT13	GSE1379	Breast cancer	Relapse Free Survival	MGH (1987-2000)	60	0.0352638	0.394251
GENE	NUDT13	E-TABM-158	Breast cancer	Disease Specific Survival	UCSF, CPMC (1989-1997)	117	0.038701	0.418732
GENE	NUDT13	GSE9195	Breast cancer	Distant Metastasis Free Survival	GUYT2	77	0.0449802	0.460562

Supplementary Table 2 continued.

GENE	NUDT13	GSE2990	Breast cancer	Distant Metastasis Free Survival	Uppsala, Oxford	54	0.0469603	0.473051
GENE	NUDT14	GSE1456-GP	Breast cancer	Overall Survival	Stockholm (1994-1996)	159	0.000239047	0.00750515
GENE	NUDT14	GSE1456-GP	Breast cancer	Disease Specific Survival	Stockholm (1994-1996)	159	0.000339345	0.0101612
GENE	NUDT14	GSE19615	Breast cancer	Distant Metastasis Free Survival	DF/HCC	115	0.00108299	0.0272457
GENE	NUDT14	GSE1456-GP	Breast cancer	Relapse Free Survival	Stockholm (1994-1996)	159	0.00779646	0.133645
GENE	NUDT14	GSE1456-GP	Breast cancer	Overall Survival	Stockholm (1994-1996)	159	0.0122938	0.18838
GENE	NUDT14	GSE3494-GP	Breast cancer	Disease Specific Survival	Uppsala (1987-1989)	236	0.012459	0.190251
GENE	NUDT14	GSE1456-GP	Breast cancer	Relapse Free Survival	Stockholm (1994-1996)	159	0.014223	0.209708
GENE	NUDT14	GSE1456-GP	Breast cancer	Disease Specific Survival	Stockholm (1994-1996)	159	0.0231649	0.296972
GENE	NUDT14	GSE9195	Breast cancer	Distant Metastasis Free Survival	GUYT2	77	0.0272372	0.331907
GENE	NUDT14	GSE9195	Breast cancer	Relapse Free Survival	GUYT2	77	0.0277202	0.335889
GENE	NUDT14	GSE6532-GP	Breast cancer	Relapse Free Survival	GUYT	87	0.0373392	0.409176
GENE	NUDT14	GSE6532-GP	Breast cancer	Distant Metastasis Free Survival	GUYT	87	0.0373392	0.409176
GENE	NUDT14	GSE4922-GP	Breast cancer	Disease Free Survival	Uppsala (1987-1989)	249	0.0408872	0.433699
GENE	NUDT14	GSE1378	Breast cancer	Relapse Free Survival	MGH (1987-2000)	60	0.0453012	0.462608
GENE	NUDT15	GSE12276	Breast cancer	Relapse Free Survival	EMC	204	0.000126287	0.00430008
GENE	NUDT15	GSE3494-GP	Breast cancer	Disease Specific Survival	Uppsala (1987-1989)	236	0.000218011	0.00692809
GENE	NUDT15	GSE4922-GP	Breast cancer	Disease Free Survival	Uppsala (1987-1989)	249	0.00182492	0.0420122
GENE	NUDT15	GSE9195	Breast cancer	Relapse Free Survival	GUYT2	77	0.00526586	0.0985749
GENE	NUDT15	GSE6532-GP	Breast cancer	Relapse Free Survival	GUYT	87	0.0117079	0.181678
GENE	NUDT15	GSE6532-GP	Breast cancer	Distant Metastasis Free Survival	GUYT	87	0.0117079	0.181678
GENE	NUDT15	GSE11121	Breast cancer	Distant Metastasis Free Survival	Mainz (1988-1998)	200	0.0181855	0.250453
GENE	NUDT15	E-TABM-158	Breast cancer	Overall Survival	UCSF, CPMC (1989-1997)	117	0.0230179	0.295663
GENE	NUDT15	E-TABM-158	Breast cancer	Relapse Free Survival	UCSF, CPMC (1989-1997)	117	0.0230179	0.295663
GENE	NUDT15	GSE7378	Breast cancer	Disease Free Survival	UCSF	54	0.0274658	0.333796
GENE	NUDT15	GSE2034	Breast cancer	Distant Metastasis Free Survival	Rotterdam (1980-1995)	286	0.0447991	0.459404
GENE	NUDT17	GSE3494-GP	Breast cancer	Disease Specific Survival	Uppsala (1987-1989)	236	4,01E+00	0.00155894
GENE	NUDT17	GSE4922-GP	Breast cancer	Disease Free Survival	Uppsala (1987-1989)	249	0.00038091	0.0112235
GENE	NUDT17	GSE12276	Breast cancer	Relapse Free Survival	EMC	204	0.00426656	0.0834938
GENE	NUDT17	GSE6532-GP	Breast cancer	Relapse Free Survival	GUYT	87	0.0317056	0.367575
GENE	NUDT17	GSE6532-GP	Breast cancer	Distant Metastasis Free Survival	GUYT	87	0.0317056	0.367575
GENE	NUDT17	GSE19615	Breast cancer	Distant Metastasis Free Survival	DF/HCC	115	0.0416468	0.438796
GENE	NUDT17	GSE1456-GP	Breast cancer	Relapse Free Survival	Stockholm (1994-1996)	159	0.0468223	0.472191
GENE	NUDT18	GSE6532-GP	Breast cancer	Relapse Free Survival	GUYT	87	0.000460512	0.0132076
GENE	NUDT18	GSE6532-GP	Breast cancer	Distant Metastasis Free Survival	GUYT	87	0.000460512	0.0132076
GENE	NUDT18	GSE3494-GP	Breast cancer	Disease Specific Survival	Uppsala (1987-1989)	236	0.000847221	0.022169
GENE	NUDT18	GSE4922-GP	Breast cancer	Disease Free Survival	Uppsala (1987-1989)	249	0.00158674	0.0374358
GENE	NUDT18	GSE12276	Breast cancer	Relapse Free Survival	EMC	204	0.00166876	0.0390271
GENE	NUDT18	GSE7390	Breast cancer	Relapse Free Survival	Uppsala, Oxford, Stockholm, IGR, GUY	198	0.00377556	0.0757535
GENE	NUDT18	GSE7390	Breast cancer	Overall Survival	Uppsala, Oxford, Stockholm, IGR, GUY	198	0.0117957	0.182689
GENE	NUDT18	GSE1378	Breast cancer	Relapse Free Survival	MGH (1987-2000)	60	0.0207155	0.274667
GENE	NUDT18	GSE19615	Breast cancer	Distant Metastasis Free Survival	DF/HCC	115	0.0231791	0.297099
GENE	NUDT18	GSE7390	Breast cancer	Distant Metastasis Free Survival	Uppsala, Oxford, Stockholm, IGR, GUY	198	0.0233396	0.298524
GENE	NUDT18	GSE11121	Breast cancer	Distant Metastasis Free Survival	Mainz (1988-1998)	200	0.029097	0.347067
GENE	NUDT18	E-TABM-158	Breast cancer	Disease Specific Survival	UCSF, CPMC (1989-1997)	117	0.0366942	0.404585
GENE	NUDT19	GSE12276	Breast cancer	Relapse Free Survival	EMC	204	5,59E+00	0.00209264
GENE	NUDT19	GSE3494-GP	Breast cancer	Disease Specific Survival	Uppsala (1987-1989)	236	0.00462811	0.0890477
GENE	NUDT19	GSE1378	Breast cancer	Relapse Free Survival	MGH (1987-2000)	60	0.012473	0.190409
GENE	NUDT19	GSE4922-GP	Breast cancer	Disease Free Survival	Uppsala (1987-1989)	249	0.0175382	0.244048
GENE	NUDT19	GSE19615	Breast cancer	Distant Metastasis Free Survival	DF/HCC	115	0.0219148	0.285719
GENE	NUDT21	GSE1456-GP	Breast cancer	Disease Specific Survival	Stockholm (1994-1996)	159	2,10E+00	0.000872756
GENE	NUDT21	GSE1456-GP	Breast cancer	Relapse Free Survival	Stockholm (1994-1996)	159	4,04E-01	0.00156754
GENE	NUDT21	GSE1456-GP	Breast cancer	Overall Survival	Stockholm (1994-1996)	159	6,94E+00	0.00253686
GENE	NUDT21	GSE1456-GP	Breast cancer	Disease Specific Survival	Stockholm (1994-1996)	159	7,87E+00	0.00283423
GENE	NUDT21	GSE3494-GP	Breast cancer	Disease Specific Survival	Uppsala (1987-1989)	236	7,96E+00	0.00286368
GENE	NUDT21	GSE12276	Breast cancer	Relapse Free Survival	EMC	204	0.000113901	0.00392734
GENE	NUDT21	GSE1456-GP	Breast cancer	Disease Specific Survival	Stockholm (1994-1996)	159	0.000139568	0.00469414
GENE	NUDT21	GSE6532-GP	Breast cancer	Relapse Free Survival	GUYT	87	0.000181314	0.00590079
GENE	NUDT21	GSE6532-GP	Breast cancer	Distant Metastasis Free Survival	GUYT	87	0.000181314	0.00590079
GENE	NUDT21	GSE1456-GP	Breast cancer	Overall Survival	Stockholm (1994-1996)	159	0.000194799	0.00628156
GENE	NUDT21	GSE9195	Breast cancer	Distant Metastasis Free Survival	GUYT2	77	0.000384715	0.0113198
GENE	NUDT21	GSE4922-GP	Breast cancer	Disease Free Survival	Uppsala (1987-1989)	249	0.000519886	0.01465
GENE	NUDT21	GSE7390	Breast cancer	Relapse Free Survival	Uppsala, Oxford, Stockholm, IGR, GUY	198	0.000569728	0.0158392
GENE	NUDT21	E-TABM-158	Breast cancer	Distant Metastasis Free Survival	UCSF, CPMC (1989-1997)	117	0.000696386	0.0187841
GENE	NUDT21	GSE1456-GP	Breast cancer	Overall Survival	Stockholm (1994-1996)	159	0.000798639	0.0210918
GENE	NUDT21	GSE7390	Breast cancer	Distant Metastasis Free Survival	Uppsala, Oxford, Stockholm, IGR, GUY	198	0.000978341	0.0250212
GENE	NUDT21	GSE7390	Breast cancer	Overall Survival	Uppsala, Oxford, Stockholm, IGR, GUY	198	0.0010995	0.0275928
GENE	NUDT21	GSE7390	Breast cancer	Relapse Free Survival	Uppsala, Oxford, Stockholm, IGR, GUY	198	0.0014017	0.0337815
GENE	NUDT21	GSE9195	Breast cancer	Relapse Free Survival	GUYT2	77	0.00155669	0.0368486
GENE	NUDT21	GSE7390	Breast cancer	Overall Survival	Uppsala, Oxford, Stockholm, IGR, GUY	198	0.00187922	0.0430372

Supplementary Table 2 continued.

GENE	NUDT21	GSE7390	Breast cancer	Distant Metastasis Free Survival	Uppsala, Oxford, Stockholm, IGR, GUY	198	0.00244358	0.0533458
GENE	NUDT21	GSE6532-GP	Breast cancer	Distant Metastasis Free Survival	GUYT	87	0.00361309	0.0731374
GENE	NUDT21	GSE6532-GP	Breast cancer	Relapse Free Survival	GUYT	87	0.00361309	0.0731374
GENE	NUDT21	GSE1379	Breast cancer	Relapse Free Survival	MGH (1987-2000)	60	0.00376121	0.0755235
GENE	NUDT21	E-TABM-158	Breast cancer	Disease Specific Survival	UCSF, CPMC (1989-1997)	117	0.00492332	0.0934985
GENE	NUDT21	GSE1378	Breast cancer	Relapse Free Survival	MGH (1987-2000)	60	0.00598932	0.109012
GENE	NUDT21	GSE7378	Breast cancer	Disease Free Survival	UCSF	54	0.00641281	0.114957
GENE	NUDT21	GSE11121	Breast cancer	Distant Metastasis Free Survival	Mainz (1988-1998)	200	0.00683997	0.120842
GENE	NUDT21	GSE12276	Breast cancer	Relapse Free Survival	EMC	204	0.00859409	0.143965
GENE	NUDT21	GSE2990	Breast cancer	Distant Metastasis Free Survival	Uppsala, Oxford	125	0.00950514	0.155397
GENE	NUDT21	GSE19615	Breast cancer	Distant Metastasis Free Survival	DF/HCC	115	0.0139667	0.206937
GENE	NUDT21	GSE2990	Breast cancer	Relapse Free Survival	Uppsala, Oxford	62	0.0139859	0.207145
GENE	NUDT21	GSE7378	Breast cancer	Disease Free Survival	UCSF	54	0.0153003	0.221166
GENE	NUDT21	GSE19615	Breast cancer	Distant Metastasis Free Survival	DF/HCC	115	0.0155257	0.223525
GENE	NUDT21	E-TABM-158	Breast cancer	Relapse Free Survival	UCSF, CPMC (1989-1997)	117	0.0186873	0.255358
GENE	NUDT21	E-TABM-158	Breast cancer	Overall Survival	UCSF, CPMC (1989-1997)	117	0.0186873	0.255358
GENE	NUDT21	GSE2990	Breast cancer	Relapse Free Survival	Uppsala, Oxford	125	0.0189221	0.257636
GENE	NUDT21	GSE7378	Breast cancer	Disease Free Survival	UCSF	54	0.0219065	0.285643
GENE	NUDT21	GSE2990	Breast cancer	Distant Metastasis Free Survival	Uppsala, Oxford	125	0.0270423	0.33029
GENE	NUDT21	GSE12276	Breast cancer	Relapse Free Survival	EMC	204	0.0288369	0.344974
GENE	NUDT21	GSE4922-GP	Breast cancer	Disease Free Survival	Uppsala (1987-1989)	249	0.0354765	0.395801
GENE	NUDT21	GSE9195	Breast cancer	Relapse Free Survival	GUYT2	77	0.0391714	0.42199
GENE	NUDT21	GSE3494-GP	Breast cancer	Disease Specific Survival	Uppsala (1987-1989)	236	0.0407309	0.432644
GENE	NUDT21	GSE11121	Breast cancer	Distant Metastasis Free Survival	Mainz (1988-1998)	200	0.0428767	0.446938
GENE	NUDT21	E-TABM-158	Breast cancer	Distant Metastasis Free Survival	UCSF, CPMC (1989-1997)	117	0.045257	0.462327
GENE	NUDT21	GSE4922-GP	Breast cancer	Disease Free Survival	Uppsala (1987-1989)	249	0.0462819	0.468807
GENE	NUDT21	GSE3143	Breast cancer	Overall Survival	Duke	158	0.0470595	0.473668
GENE	NUDT22	GSE4922-GP	Breast cancer	Disease Free Survival	Uppsala (1987-1989)	249	0.00251691	0.0546438
GENE	NUDT22	GSE1456-GP	Breast cancer	Relapse Free Survival	Stockholm (1994-1996)	159	0.00508309	0.0958776
GENE	NUDT22	GSE9195	Breast cancer	Distant Metastasis Free Survival	GUYT2	77	0.0202395	0.270206
GENE	NUDT22	GSE1456-GP	Breast cancer	Overall Survival	Stockholm (1994-1996)	159	0.020787	0.275332
GENE	NUDT22	GSE1456-GP	Breast cancer	Disease Specific Survival	Stockholm (1994-1996)	159	0.024009	0.304424
GENE	NUDT22	GSE1379	Breast cancer	Relapse Free Survival	MGH (1987-2000)	60	0.0288142	0.344791
GENE	NUDT22	GSE12276	Breast cancer	Relapse Free Survival	EMC	204	0.0316612	0.367233
GENE	NUDT22	GSE3494-GP	Breast cancer	Disease Specific Survival	Uppsala (1987-1989)	236	0.036739	0.404905
GENE	NUDT3	GSE3494-GP	Breast cancer	Disease Specific Survival	Uppsala (1987-1989)	236	9,56E-01	0.000428924
GENE	NUDT3	GSE12276	Breast cancer	Relapse Free Survival	EMC	204	2,86E+00	0.00114985
GENE	NUDT3	GSE4922-GP	Breast cancer	Disease Free Survival	Uppsala (1987-1989)	249	0.00103224	0.0261723
GENE	NUDT3	GSE3143	Breast cancer	Overall Survival	Duke	158	0.00111604	0.0279394
GENE	NUDT3	GSE7390	Breast cancer	Relapse Free Survival	Uppsala, Oxford, Stockholm, IGR, GUY	198	0.00229295	0.0506515
GENE	NUDT3	GSE11121	Breast cancer	Distant Metastasis Free Survival	Mainz (1988-1998)	200	0.00400309	0.0793702
GENE	NUDT3	GSE6532-GP	Breast cancer	Distant Metastasis Free Survival	GUYT	87	0.00487558	0.0927836
GENE	NUDT3	GSE6532-GP	Breast cancer	Relapse Free Survival	GUYT	87	0.00487558	0.0927836
GENE	NUDT3	E-TABM-158	Breast cancer	Disease Specific Survival	UCSF, CPMC (1989-1997)	117	0.00642807	0.115169
GENE	NUDT3	E-TABM-158	Breast cancer	Relapse Free Survival	UCSF, CPMC (1989-1997)	117	0.00699605	0.122965
GENE	NUDT3	E-TABM-158	Breast cancer	Overall Survival	UCSF, CPMC (1989-1997)	117	0.00699605	0.122965
GENE	NUDT3	GSE7378	Breast cancer	Disease Free Survival	UCSF	54	0.00722013	0.12599
GENE	NUDT3	GSE1456-GP	Breast cancer	Relapse Free Survival	Stockholm (1994-1996)	159	0.00739526	0.128335
GENE	NUDT3	GSE7378	Breast cancer	Disease Free Survival	UCSF	54	0.00775536	0.133105
GENE	NUDT3	GSE2990	Breast cancer	Relapse Free Survival	Uppsala, Oxford	62	0.0116751	0.181299
GENE	NUDT3	GSE1379	Breast cancer	Relapse Free Survival	MGH (1987-2000)	60	0.0133945	0.200682
GENE	NUDT3	GSE7378	Breast cancer	Disease Free Survival	UCSF	54	0.0135017	0.201862
GENE	NUDT3	GSE9195	Breast cancer	Distant Metastasis Free Survival	GUYT2	77	0.013604	0.202983
GENE	NUDT3	GSE9195	Breast cancer	Relapse Free Survival	GUYT2	77	0.0160833	0.229307
GENE	NUDT3	GSE19615	Breast cancer	Distant Metastasis Free Survival	DF/HCC	115	0.016532	0.233906
GENE	NUDT3	GSE7378	Breast cancer	Disease Free Survival	UCSF	54	0.0205623	0.273236
GENE	NUDT3	GSE7390	Breast cancer	Distant Metastasis Free Survival	Uppsala, Oxford, Stockholm, IGR, GUY	198	0.0293146	0.34881
GENE	NUDT3	GSE12276	Breast cancer	Relapse Free Survival	EMC	204	0.0304298	0.357651
GENE	NUDT3	GSE2990	Breast cancer	Distant Metastasis Free Survival	Uppsala, Oxford	54	0.0327768	0.375757
GENE	NUDT3	GSE7849	Breast cancer	Disease Free Survival	Duke (1990-2001)	76	0.0337824	0.383318
GENE	NUDT3	GSE9195	Breast cancer	Distant Metastasis Free Survival	GUYT2	77	0.0339613	0.384651
GENE	NUDT3	GSE1456-GP	Breast cancer	Overall Survival	Stockholm (1994-1996)	159	0.03542	0.39539
GENE	NUDT3	GSE9195	Breast cancer	Relapse Free Survival	GUYT2	77	0.0389642	0.420557
GENE	NUDT3	GSE7390	Breast cancer	Overall Survival	Uppsala, Oxford, Stockholm, IGR, GUY	198	0.0431851	0.448959
GENE	NUDT3	GSE2034	Breast cancer	Distant Metastasis Free Survival	Rotterdam (1980-1995)	286	0.0498326	0.490618

Supplementary Table 2 continued.

GENE	NUDT4	GSE3494-GP	Breast cancer	Disease Specific Survival	Uppsala (1987-1989)	236	1,72E+00	0.000730844
GENE	NUDT4	GSE4922-GP	Breast cancer	Disease Free Survival	Uppsala (1987-1989)	249	2,44E+00	0.000997958
GENE	NUDT4	GSE1456-GP	Breast cancer	Disease Specific Survival	Stockholm (1994-1996)	159	4,41E+00	0.00169406
GENE	NUDT4	GSE3494-GP	Breast cancer	Disease Specific Survival	Uppsala (1987-1989)	236	4,53E+00	0.0017369
GENE	NUDT4	GSE1456-GP	Breast cancer	Relapse Free Survival	Stockholm (1994-1996)	159	0.000124684	0.00425213
GENE	NUDT4	GSE4922-GP	Breast cancer	Disease Free Survival	Uppsala (1987-1989)	249	0.000163224	0.00538343
GENE	NUDT4	GSE4922-GP	Breast cancer	Disease Free Survival	Uppsala (1987-1989)	249	0.000171874	0.00563179
GENE	NUDT4	GSE1456-GP	Breast cancer	Overall Survival	Stockholm (1994-1996)	159	0.000472083	0.013491
GENE	NUDT4	GSE3494-GP	Breast cancer	Disease Specific Survival	Uppsala (1987-1989)	236	0.000603177	0.0166272
GENE	NUDT4	GSE7390	Breast cancer	Overall Survival	Uppsala, Oxford, Stockholm, IGR, GUY	198	0.000612946	0.0168559
GENE	NUDT4	GSE7390	Breast cancer	Relapse Free Survival	Uppsala, Oxford, Stockholm, IGR, GUY	198	0.000646552	0.0176378
GENE	NUDT4	GSE7390	Breast cancer	Distant Metastasis Free Survival	Uppsala, Oxford, Stockholm, IGR, GUY	198	0.000691576	0.0186741
GENE	NUDT4	GSE12093	Breast cancer	Distant Metastasis Free Survival	IO, NCI, TUM, CCF (1992-2000)	136	0.000777208	0.0206128
GENE	NUDT4	GSE9195	Breast cancer	Distant Metastasis Free Survival	GUYT2	77	0.000945281	0.0243092
GENE	NUDT4	GSE7390	Breast cancer	Overall Survival	Uppsala, Oxford, Stockholm, IGR, GUY	198	0.00105321	0.026617
GENE	NUDT4	GSE9893	Breast cancer	Overall Survival	Montpellier, Bordeaux, Turin (1989-20	155	0.00112068	0.0280366
GENE	NUDT4	GSE7390	Breast cancer	Distant Metastasis Free Survival	Uppsala, Oxford, Stockholm, IGR, GUY	198	0.00113929	0.0284252
GENE	NUDT4	GSE11121	Breast cancer	Distant Metastasis Free Survival	Mainz (1988-1998)	200	0.00142585	0.0342638
GENE	NUDT4	GSE3494-GP	Breast cancer	Disease Specific Survival	Uppsala (1987-1989)	236	0.00169141	0.0394636
GENE	NUDT4	GSE12093	Breast cancer	Distant Metastasis Free Survival	IO, NCI, TUM, CCF (1992-2000)	136	0.00227419	0.0503132
GENE	NUDT4	E-TABM-158	Breast cancer	Disease Specific Survival	UCSF, CPMC (1989-1997)	117	0.0026015	0.0561303
GENE	NUDT4	GSE9195	Breast cancer	Distant Metastasis Free Survival	GUYT2	77	0.00338458	0.0694084
GENE	NUDT4	GSE7390	Breast cancer	Relapse Free Survival	Uppsala, Oxford, Stockholm, IGR, GUY	198	0.00342565	0.070083
GENE	NUDT4	GSE7390	Breast cancer	Relapse Free Survival	Uppsala, Oxford, Stockholm, IGR, GUY	198	0.00347544	0.0708983
GENE	NUDT4	GSE4922-GP	Breast cancer	Disease Free Survival	Uppsala (1987-1989)	249	0.00486642	0.0926462
GENE	NUDT4	GSE7390	Breast cancer	Relapse Free Survival	Uppsala, Oxford, Stockholm, IGR, GUY	198	0.00664542	0.118175
GENE	NUDT4	GSE2034	Breast cancer	Distant Metastasis Free Survival	Rotterdam (1980-1995)	286	0.00748795	0.129569
GENE	NUDT4	GSE7390	Breast cancer	Overall Survival	Uppsala, Oxford, Stockholm, IGR, GUY	198	0.00876238	0.146104
GENE	NUDT4	GSE2034	Breast cancer	Distant Metastasis Free Survival	Rotterdam (1980-1995)	286	0.00890433	0.147899
GENE	NUDT4	GSE9195	Breast cancer	Distant Metastasis Free Survival	GUYT2	77	0.0115446	0.179789
GENE	NUDT4	GSE12276	Breast cancer	Relapse Free Survival	EMC	204	0.0120903	0.186064
GENE	NUDT4	GSE3494-GP	Breast cancer	Disease Specific Survival	Uppsala (1987-1989)	236	0.012228	0.187633
GENE	NUDT4	GSE1456-GP	Breast cancer	Overall Survival	Stockholm (1994-1996)	159	0.0128291	0.19441
GENE	NUDT4	GSE2990	Breast cancer	Distant Metastasis Free Survival	Uppsala, Oxford	54	0.0128856	0.19504
GENE	NUDT4	GSE2990	Breast cancer	Relapse Free Survival	Uppsala, Oxford	62	0.0132153	0.198705
GENE	NUDT4	GSE1456-GP	Breast cancer	Disease Specific Survival	Stockholm (1994-1996)	159	0.013833	0.205484
GENE	NUDT4	GSE2990	Breast cancer	Relapse Free Survival	Uppsala, Oxford	62	0.0143533	0.211111
GENE	NUDT4	GSE9195	Breast cancer	Relapse Free Survival	GUYT2	77	0.0143878	0.211481
GENE	NUDT4	GSE9195	Breast cancer	Relapse Free Survival	GUYT2	77	0.0151784	0.219885
GENE	NUDT4	GSE7390	Breast cancer	Distant Metastasis Free Survival	Uppsala, Oxford, Stockholm, IGR, GUY	198	0.0158704	0.227109
GENE	NUDT4	E-TABM-158	Breast cancer	Overall Survival	UCSF, CPMC (1989-1997)	117	0.0159618	0.228053
GENE	NUDT4	E-TABM-158	Breast cancer	Relapse Free Survival	UCSF, CPMC (1989-1997)	117	0.0159618	0.228053
GENE	NUDT4	E-TABM-158	Breast cancer	Disease Specific Survival	UCSF, CPMC (1989-1997)	117	0.017148	0.240142
GENE	NUDT4	E-TABM-158	Breast cancer	Distant Metastasis Free Survival	UCSF, CPMC (1989-1997)	117	0.0174498	0.243165
GENE	NUDT4	E-TABM-158	Breast cancer	Relapse Free Survival	UCSF, CPMC (1989-1997)	117	0.0179843	0.248473
GENE	NUDT4	E-TABM-158	Breast cancer	Overall Survival	UCSF, CPMC (1989-1997)	117	0.0179843	0.248473
GENE	NUDT4	GSE7390	Breast cancer	Distant Metastasis Free Survival	Uppsala, Oxford, Stockholm, IGR, GUY	198	0.0184241	0.252792
GENE	NUDT4	GSE1456-GP	Breast cancer	Relapse Free Survival	Stockholm (1994-1996)	159	0.0214813	0.281754
GENE	NUDT4	GSE7378	Breast cancer	Disease Free Survival	UCSF	54	0.0246179	0.309728
GENE	NUDT4	GSE9195	Breast cancer	Relapse Free Survival	GUYT2	77	0.0250001	0.313028
GENE	NUDT4	GSE1456-GP	Breast cancer	Relapse Free Survival	Stockholm (1994-1996)	159	0.0259464	0.321103
GENE	NUDT4	GSE9195	Breast cancer	Distant Metastasis Free Survival	GUYT2	77	0.0267098	0.327521
GENE	NUDT4	GSE2990	Breast cancer	Relapse Free Survival	Uppsala, Oxford	125	0.0275034	0.334105
GENE	NUDT4	GSE1456-GP	Breast cancer	Disease Specific Survival	Stockholm (1994-1996)	159	0.0281044	0.339033
GENE	NUDT4	GSE19615	Breast cancer	Distant Metastasis Free Survival	DF/HCC	115	0.0281572	0.339464
GENE	NUDT4	E-TABM-158	Breast cancer	Relapse Free Survival	UCSF, CPMC (1989-1997)	117	0.029298	0.348677
GENE	NUDT4	E-TABM-158	Breast cancer	Overall Survival	UCSF, CPMC (1989-1997)	117	0.029298	0.348677
GENE	NUDT4	E-TABM-158	Breast cancer	Disease Specific Survival	UCSF, CPMC (1989-1997)	117	0.0317479	0.3679
GENE	NUDT4	GSE9195	Breast cancer	Distant Metastasis Free Survival	GUYT2	77	0.0329196	0.376837
GENE	NUDT4	GSE7390	Breast cancer	Relapse Free Survival	Uppsala, Oxford, Stockholm, IGR, GUY	198	0.0337058	0.382746
GENE	NUDT4	GSE1456-GP	Breast cancer	Overall Survival	Stockholm (1994-1996)	159	0.0337077	0.38276
GENE	NUDT4	GSE1456-GP	Breast cancer	Relapse Free Survival	Stockholm (1994-1996)	159	0.0341123	0.385774
GENE	NUDT4	GSE6532-GP	Breast cancer	Distant Metastasis Free Survival	GUYT	87	0.0344999	0.388643
GENE	NUDT4	GSE6532-GP	Breast cancer	Relapse Free Survival	GUYT	87	0.0344999	0.388643
GENE	NUDT4	GSE7378	Breast cancer	Disease Free Survival	UCSF	54	0.0350126	0.392414
GENE	NUDT4	GSE6532-GP	Breast cancer	Distant Metastasis Free Survival	GUYT	87	0.0354423	0.395553
GENE	NUDT4	GSE6532-GP	Breast cancer	Relapse Free Survival	GUYT	87	0.0354423	0.395553
GENE	NUDT4	GSE7390	Breast cancer	Overall Survival	Uppsala, Oxford, Stockholm, IGR, GUY	198	0.0356565	0.39711
GENE	NUDT4	GSE12276	Breast cancer	Relapse Free Survival	EMC	204	0.0379446	0.413446
GENE	NUDT4	GSE2990	Breast cancer	Relapse Free Survival	Uppsala, Oxford	125	0.0394757	0.424087

Supplementary Table 2 continued.

GENE	NUDT4	GSE12093	Breast cancer	Distant Metastasis Free Survival	IO, NCI, TUM, CCF (1992-2000)	136	0.0414617	0.437558
GENE	NUDT4	GSE7378	Breast cancer	Disease Free Survival	UCSF	54	0.0425196	0.444588
GENE	NUDT4	GSE7390	Breast cancer	Overall Survival	Uppsala, Oxford, Stockholm, IGR, GUY	198	0.0446557	0.458484
GENE	NUDT4	E-TABM-158	Breast cancer	Distant Metastasis Free Survival	UCSF, CPMC (1989-1997)	117	0.0470834	0.473816
GENE	NUDT4	GSE1378	Breast cancer	Relapse Free Survival	MGH (1987-2000)	60	0.0490205	0.485715
GENE	NUDT4	GSE4922-GP	Breast cancer	Disease Free Survival	Uppsala (1987-1989)	249	0.04905	0.485895
GENE	NUDT4	GSE11121	Breast cancer	Distant Metastasis Free Survival	Mainz (1988-1998)	200	0.0491307	0.486384
GENE	NUDT6	E-TABM-158	Breast cancer	Overall Survival	UCSF, CPMC (1989-1997)	117	2,66E-01	0.00107743
GENE	NUDT6	E-TABM-158	Breast cancer	Relapse Free Survival	UCSF, CPMC (1989-1997)	117	2,66E-01	0.00107743
GENE	NUDT6	E-TABM-158	Breast cancer	Disease Specific Survival	UCSF, CPMC (1989-1997)	117	0.000274366	0.00845715
GENE	NUDT6	E-TABM-158	Breast cancer	Distant Metastasis Free Survival	UCSF, CPMC (1989-1997)	117	0.000427997	0.0124046
GENE	NUDT6	GSE3494-GP	Breast cancer	Disease Specific Survival	Uppsala (1987-1989)	236	0.000752018	0.0200467
GENE	NUDT6	GSE4922-GP	Breast cancer	Disease Free Survival	Uppsala (1987-1989)	249	0.0017522	0.0406291
GENE	NUDT6	GSE11121	Breast cancer	Distant Metastasis Free Survival	Mainz (1988-1998)	200	0.00196096	0.0445684
GENE	NUDT6	GSE19615	Breast cancer	Distant Metastasis Free Survival	DF/HCC	115	0.00746022	0.1292
GENE	NUDT6	GSE1456-GP	Breast cancer	Overall Survival	Stockholm (1994-1996)	159	0.0086986	0.145295
GENE	NUDT6	GSE3494-GP	Breast cancer	Disease Specific Survival	Uppsala (1987-1989)	236	0.0115402	0.179738
GENE	NUDT6	GSE1379	Breast cancer	Relapse Free Survival	MGH (1987-2000)	60	0.0170319	0.238973
GENE	NUDT6	GSE12276	Breast cancer	Relapse Free Survival	EMC	204	0.01722	0.240865
GENE	NUDT6	GSE12276	Breast cancer	Relapse Free Survival	EMC	204	0.0214025	0.281029
GENE	NUDT6	GSE1456-GP	Breast cancer	Relapse Free Survival	Stockholm (1994-1996)	159	0.0230284	0.295756
GENE	NUDT6	GSE2990	Breast cancer	Relapse Free Survival	Uppsala, Oxford	125	0.0344012	0.387914
GENE	NUDT6	GSE4922-GP	Breast cancer	Disease Free Survival	Uppsala (1987-1989)	249	0.0466929	0.471382
GENE	NUDT6	GSE19615	Breast cancer	Distant Metastasis Free Survival	DF/HCC	115	0.0478059	0.478289
GENE	NUDT7	GSE12276	Breast cancer	Relapse Free Survival	EMC	204	2,17E-01	0.000111147
GENE	NUDT7	GSE1379	Breast cancer	Relapse Free Survival	MGH (1987-2000)	60	1,22E+00	0.000535866
GENE	NUDT7	GSE7390	Breast cancer	Relapse Free Survival	Uppsala, Oxford, Stockholm, IGR, GUY	198	0.000290184	0.0088773
GENE	NUDT7	GSE7390	Breast cancer	Distant Metastasis Free Survival	Uppsala, Oxford, Stockholm, IGR, GUY	198	0.000380512	0.0112135
GENE	NUDT7	GSE9195	Breast cancer	Distant Metastasis Free Survival	GUYT2	77	0.000795474	0.0210212
GENE	NUDT7	E-TABM-158	Breast cancer	Relapse Free Survival	UCSF, CPMC (1989-1997)	117	0.00218146	0.0486317
GENE	NUDT7	E-TABM-158	Breast cancer	Overall Survival	UCSF, CPMC (1989-1997)	117	0.00218146	0.0486317
GENE	NUDT7	GSE7390	Breast cancer	Overall Survival	Uppsala, Oxford, Stockholm, IGR, GUY	198	0.00229334	0.0506585
GENE	NUDT7	E-TABM-158	Breast cancer	Disease Specific Survival	UCSF, CPMC (1989-1997)	117	0.00258982	0.0559257
GENE	NUDT7	GSE7390	Breast cancer	Relapse Free Survival	Uppsala, Oxford, Stockholm, IGR, GUY	198	0.00709311	0.124279
GENE	NUDT7	GSE11121	Breast cancer	Distant Metastasis Free Survival	Mainz (1988-1998)	200	0.00721944	0.125981
GENE	NUDT7	E-TABM-158	Breast cancer	Distant Metastasis Free Survival	UCSF, CPMC (1989-1997)	117	0.00802581	0.136644
GENE	NUDT7	GSE1456-GP	Breast cancer	Relapse Free Survival	Stockholm (1994-1996)	159	0.00878141	0.146346
GENE	NUDT7	GSE9893	Breast cancer	Overall Survival	Montpellier, Bordeaux, Turin (1989-20	155	0.00909411	0.150284
GENE	NUDT7	GSE19615	Breast cancer	Distant Metastasis Free Survival	DF/HCC	115	0.00912021	0.150611
GENE	NUDT7	GSE9195	Breast cancer	Relapse Free Survival	GUYT2	77	0.0113827	0.177908
GENE	NUDT7	GSE9195	Breast cancer	Relapse Free Survival	GUYT2	77	0.0129318	0.195556
GENE	NUDT7	GSE12093	Breast cancer	Distant Metastasis Free Survival	IO, NCI, TUM, CCF (1992-2000)	136	0.0131926	0.198453
GENE	NUDT7	GSE9195	Breast cancer	Distant Metastasis Free Survival	GUYT2	77	0.0137325	0.204388
GENE	NUDT7	GSE3494-GP	Breast cancer	Disease Specific Survival	Uppsala (1987-1989)	236	0.0170886	0.239545
GENE	NUDT7	GSE19615	Breast cancer	Distant Metastasis Free Survival	DF/HCC	115	0.020487	0.272531
GENE	NUDT7	GSE1456-GP	Breast cancer	Disease Specific Survival	Stockholm (1994-1996)	159	0.0247139	0.310559
GENE	NUDT7	GSE1456-GP	Breast cancer	Overall Survival	Stockholm (1994-1996)	159	0.0256933	0.318957
GENE	NUDT7	GSE1456-GP	Breast cancer	Overall Survival	Stockholm (1994-1996)	159	0.0321577	0.371044
GENE	NUDT7	GSE12276	Breast cancer	Relapse Free Survival	EMC	204	0.0331936	0.378905
GENE	NUDT7	GSE2990	Breast cancer	Relapse Free Survival	Uppsala, Oxford	125	0.0368291	0.405549
GENE	NUDT7	E-TABM-158	Breast cancer	Distant Metastasis Free Survival	UCSF, CPMC (1989-1997)	117	0.0387761	0.419253
GENE	NUDT7	GSE9195	Breast cancer	Relapse Free Survival	GUYT2	77	0.0399277	0.427185
GENE	NUDT7	E-TABM-158	Breast cancer	Disease Specific Survival	UCSF, CPMC (1989-1997)	117	0.0424203	0.443932
GENE	NUDT7	GSE3494-GP	Breast cancer	Disease Specific Survival	Uppsala (1987-1989)	236	0.0430055	0.447783
GENE	NUDT7	GSE6532-GP	Breast cancer	Relapse Free Survival	GUYT	87	0.0442021	0.455566
GENE	NUDT7	GSE6532-GP	Breast cancer	Distant Metastasis Free Survival	GUYT	87	0.0442021	0.455566
GENE	NUDT8	GSE1378	Breast cancer	Relapse Free Survival	MGH (1987-2000)	60	0.000828304	0.0217509
GENE	NUDT8	GSE9893	Breast cancer	Overall Survival	Montpellier, Bordeaux, Turin (1989-20	155	0.0311734	0.363459
GENE	NUDT8	GSE1379	Breast cancer	Relapse Free Survival	MGH (1987-2000)	60	0.0456371	0.46474
GENE	NUDT9	GSE9893	Breast cancer	Overall Survival	Montpellier, Bordeaux, Turin (1989-20	155	4,87E+00	0.00185045
GENE	NUDT9	GSE1456-GP	Breast cancer	Relapse Free Survival	Stockholm (1994-1996)	159	0.00032754	0.00985571
GENE	NUDT9	GSE2034	Breast cancer	Distant Metastasis Free Survival	Rotterdam (1980-1995)	286	0.00113149	0.0282624
GENE	NUDT9	GSE11121	Breast cancer	Distant Metastasis Free Survival	Mainz (1988-1998)	200	0.00126977	0.031116
GENE	NUDT9	GSE1456-GP	Breast cancer	Disease Specific Survival	Stockholm (1994-1996)	159	0.00348886	0.0711176
GENE	NUDT9	GSE7390	Breast cancer	Relapse Free Survival	Uppsala, Oxford, Stockholm, IGR, GUY	198	0.00442119	0.0858835
GENE	NUDT9	GSE1456-GP	Breast cancer	Overall Survival	Stockholm (1994-1996)	159	0.00510917	0.0962641
GENE	NUDT9	GSE12276	Breast cancer	Relapse Free Survival	EMC	204	0.0126854	0.1928
GENE	NUDT9	GSE7390	Breast cancer	Distant Metastasis Free Survival	Uppsala, Oxford, Stockholm, IGR, GUY	198	0.0133766	0.200486
GENE	NUDT9	GSE9195	Breast cancer	Distant Metastasis Free Survival	GUYT2	77	0.0195727	0.263887
